# Twelve tips for promoting professionalism through reflective small group learning

**DOI:** 10.15694/mep.2017.000043

**Published:** 2017-03-06

**Authors:** Hilary Neve, Rachel Morris

**Affiliations:** 1 Peninsula School of Medicine, Plymouth University, Plymouth, UK; 2 School of Clinical Medicine, University of Cambridge, Cambridge, UK

**Keywords:** Professionalism, small group learning, facilitation, experiential learning, reflection

## Abstract

This article was migrated. The article was marked as recommended.

The importance of placing small group learning at the heart of professionalism curricula is increasingly being recognised within undergraduate medical education. Facilitated small groups provide a valuable setting for students to reflect upon their experiences and to learn about broad aspects of professionalism. By better understanding relevant learning theory and evidence, we can identify approaches for increasing the effectiveness of such groups and overcoming potential barriers to learning. Training facilitators is a vital part of this process. The following tips are based on the literature and the authors’ experiences of leading small group learning programmes in two UK medical schools. They provide guidance and ideas for designing an experiential small group learning programmed focused on professionalism and for supporting and training a team to facilitate effective learning in this setting.

## Introduction

While there is broad agreement that medical education curricula should support and develop students’ professionalism, there is ongoing debate both as to the definition of professionalism (e.g Cohen 2006, van Mook et al. 2009, Hodges et al. 2011) and how best to teach it (e.g. Howe et al. 2002, Birden et al. 2013). In their systematic review, Passi et al conclude that professionalism in medicine is *“an extraordinarily complex phenomenon”* (2012 p25) and that medical education should provide multiple learning opportunities for students to experience and reflect, over time, on the underlying concepts. The important role of professionalism programmes in supporting students’ professional identity formation, is also well recognised, enabling them to think, feel and act like a doctor within a community of practice (Cruess & Cruess 2014).

Learning effectively from one’s experience is critical in developing and maintaining lifelong competence ( Mann 2009). Whilst many medical students gain clinical experiences early in their undergraduate course, experience itself does not necessarily lead to learning ( Epstein & Hundert 2002). Students benefit from opportunities and “ *dedicated pauses*” ( Ho 2015 p 1290) to share, reflect and make sense of their experiences. Critical reflection can enable students to integrate new learning with existing knowledge, to understand their own beliefs, attitudes and values and to consider and integrate the emotional aspects of their learning. Reflecting within facilitated small groups, rather than alone, can be particularly effective; small groups can provide mutual support, offer information from multiple perspectives, and challenge students to consider things more deeply ( Platzer 2000). Collaborative reflection can also prepare students for participation in interprofessional teams ( Mann 2009).

Candid discussion within a safe community of learners can support students’ moral development, enable them to stay true to their values ( Rabow et al 2010), develop their professional identity ( Wald 2015) and may reduce the ethical erosion that can occur as students progress through their medical programme ( Feudtner et al 1994). Storytelling and reflecting on clinical experiences in small groups can enhance students’ ability to confront uncertainty and deal with the complexity of the real world ( Fraser & Greenhalgh 2001, Neve et al. 2016), help students develop self-awareness (Benbassat & Baumal 2005), creativity in communication ( Salmon & Young 2011) and cultivate and sustain curiosity, vital for understanding each patient’s unique experience of illness ( Dyche & Epstein 2011).

The authors share here lessons learnt from a total of 18 years’ experience of leading professionalism small group learning programmes in two very different UK medical schools. The tips are designed to be useful for programme leads and small group facilitators.

## Tip 1: Use student’s own significant experiences, stories and dilemmas as a starting point

Student experiences need to be the focus of small group professionalism sessions. It is important to encourage students to tell their stories in their own and their patients’ words, rather than the structured medicalised histories, devoid of emotions and social context often used in traditional clinical presentations ( Rosenbaum et al. 2005). Narrative can trigger the exploration of a range of professionalism issues and encourage students to consider a patient’s issues holistically, to develop empathy and discern meaning ( Greenhalgh & Hurwitz 1999).

Students may need direction at first to decide which stories to share. Complex problems are particularly helpful in stimulating reflective thinking ( Mann 2009). Stories with an emotional component, or which leave questions unanswered often produce deep discussion and challenge students’ thinking, activating the ‘right brain’ and connecting theory with feelings. By encouraging students to reflect on ‘what to share’ before the session, for example posting on the group’s e-discussion board, facilitators and students can see emergent themes and prepare for the session. As students progress, they should be expected to develop their thinking prior to the group session and consider ‘how the group might help me better understand this’.

Students value sharing their experiences in groups, recognising that exploring complex real life situations rather than the “ *superficial or trivial*’ scenarios used in didactic teaching sessions, can be the best way to learn professionalism ( Birden & Usherwood 2013, P407). In learning to tell stories, students may also learn to listen better to others’ stories (Prosnky et al. 2004) and accept and appreciate others’ perspectives. Reflection on critical events also prepares students for their future quality improvement roles, where reviewing and learning from critical incidents is an important element of patient safety.

## Tip 2: Balance the student agenda with your professionalism curricula goals

Small group discussions can also address professionalism learning outcomes, including those required by national accrediting bodies. Curricula need to be flexible and respond to changing understandings of professionalism. Managing the balance between school and student agendas requires considerable expertise; students will resist if sessions become focused on school outcomes, while important professionalism issues may be missed if students do not bring relevant stories to the group.

Skilled facilitators can support students to meet both agendas by identifying and highlighting connections between student stories and curriculum goals. Alternatively they can give each session a broad professionalism theme, guiding students to look out for and bring relevant experiences (such as an example of good team working or shared decision making) to the session. Monitoring the issues raised by students in different year groups and designing the professionalism curriculum to map onto these, can help ensure that learning outcomes align with students’ learning experiences at each stage of the programme.

Sessions also need to be flexible: opportunities for students to go ‘off piste’ at times and share ‘burning issues’, whether relevant to the session theme or not, can be one of the most valuable parts of group learning. Electronic discussion boards, running alongside group sessions, can extend opportunities for group discussion.

## Tip 3: Use small groups as a springboard for developing professional skills

Cooperative learning activities, such as small groups, can help students develop vital interpersonal ( Prince 2004) and workplace skills. In the first year, students may need to learn how to confidently speak in a group, share their views and how to come to sessions well prepared. As they progress, they should be expected, with guidance, to develop and demonstrate higher order skills, such as the ability to critically reflect and to give and respond to constructive feedback. In later years, students should demonstrate the ability to lead sessions and to evaluate and work together as a group to improve group functioning. Explicitly defining and assessing the skills expected of students can guide both learners and facilitators, directing students’ learning as well as demonstrating additional benefits of the programme. Incorporating self, peer and facilitator feedback can help students identify ways they can further improve their professionalism.

Students often best learn critical reflection skills through discussion with others ( Baernstein and Fryer-Edwards 2003). Skilled facilitators play an important part in role modelling and helping students develop these skills. They can use a range of approaches to do so, for example, using probing questions, incorporating end of session reflective templates or the processing cycle ( Midmer 2002) to promote reflection and using drawing or role play to help students see or consider professionalism experiences from a new perspective. Incorporating a 10 minute evaluation slot into the end of each session encourages students to consider what they have learnt. It can be useful to record concrete action points, such as ‘what I will do differently’ or ‘my next step will be..’

## Tip 4: Use appropriate techniques to challenge students

Some students see professionalism as a ‘soft’ subject, enjoy chatting about it but resist facilitator attempts to stretch them and make discussions more robust. Students may not always appreciate the value of what they are learning, particularly where, once grasped, it may sometimes seem like ‘common sense’. Facilitators can help students appreciate the intellectual challenges of professionalism by encouraging them to research and link experiences to knowledge, theoretical models and national guidance. Challenging them to go beyond discussion to make decisions about dilemmas and difficult scenarios can demonstrate the importance of using evidence, guidance and ethical principles in practice. Similarly, encouraging students to rehearse interactions with patients and colleagues through role play, can help them appreciate that being professional is not as easy as it may initially seem. Asking students what a senior trusted colleague would do, or what advice they would give their future selves can help increase the level of challenge without being confrontational.

## Tip 5: Balance challenge with support

Students will develop best when challenge takes place in a safe, supportive environment ( Daloz 1986). Facilitators need to help the group agree and use group rules, such as confidentiality and respect, to ensure that students feel comfortable and confident to express opinions safely, share professionalism difficulties and dilemmas and to try things out. A growing literature highlights the important role of emotions in developing professionalism, ensuring clinicians become “ *humane healers*” ( Guillemin 2015 p726). The transition from student to young doctor, in particular, can provoke considerable negative emotion ( Monrouxe et al. 2015). Medical students are often embarrassed or uncomfortable when talking about their own and others’ emotional responses. Our experience is that encouraging students to share personal and family experiences of ill-health or healthcare often brings important learning for the group. Students need to feel safe to share these issues and to practice skills such as expressing emotion. Facilitators can help by sharing problems that they themselves are grappling with and modelling emotional talk. This can have a powerful impact on learners.

## Tip 6: Be alert to the hidden curriculum and the power of role modelling

The hidden curriculum is well recognised as a powerful influence on the development of students’ professionalism and identity (Hafferty 1995). In the small group context the hidden curriculum may impact on students in two particular ways. Firstly they may hear negative views about small group learning, reflection and professionalism learning from other students and staff. Secondly, students may have experiences, often of clinical role models, which either conflict with or complement the learning in the formal professionalism curriculum. Students can struggle profoundly with clinical experiences which clash with the professional values that they have been taught. The small group setting can support students to question these experiences (‘What was it exactly that I didn’t agree with?’, ‘What could ***I*** do in a similar situation?’). Alerting students to the existence of the hidden curriculum and promoting discussion of its benefits and risks can be very helpful for students (Neve & Collett 2014). Students often take positive experiences for granted and it is important to encourage conscious analysis of these (‘What exactly did he/she do well?’), helping them reflect on the kind of doctor they want to become and the attributes they need to develop.

The accepted ‘medical culture’ and the unprofessional behaviour of clinicians can undermine students’ perceptions of professionalism learning. Unless faculty are seen to act on student feedback and challenge unprofessional behaviour, students can become disengaged and disillusioned ( Wood 2016).

## Tip 7: Demonstrate relevance and application to future clinical practice

It is often hard for students, particularly in early years, to project forwards to see the relevance of their experiences to their future roles and to consider what they might find difficult. For example, early years’ students may struggle with the concept of patient safety if they have never experienced medical error in practice. By sharing their own stories, clinical facilitators can contextualise students’ learning and highlight why issues are important. However they need do so thoughtfully, so as not to hijack or over-direct group discussions. The use of quotes from recently qualified doctors, or the findings of preparedness for practice research ( Kellett 2015), can also help students see the relevance and imminent clinical reality of their professionalism learning.

To work in today’s complex world, students must adapt and apply their learning to each unique situation, similar or different ( Fraser and Greenhalgh 2001)). Transfer of learning is hard to achieve but can be facilitated by active problem-solving and practice with multiple dissimilar problems ( Norman 2009). Facilitators can encourage students to compare and contrast their different experiences (‘What is the same?, what is different?’) and use hypothetical questions (‘But what if?.’). They can also ask students to apply their learning to new professionalism dilemmas or to their next placement, perhaps reflecting on this on their group e-discussion board.

## Tip 8: Give students time and freedom to define professionalism themselves

As already discussed, there have been numerous attempts to define professionalism, although there is broad agreement as to many of its elements. Our approach has been to ensure students are exposed to the important issues, while not always explicitly labelling them as ‘professionalism’. This approach is supported by research showing that students who had early interaction with patients and opportunities to reflect on, and make sense of these through conversations about professionalism in small groups, developed more nuanced and complex understandings of professionalism than those where professionalism was taught predominately through lectures ( Monrouxe et al. 2011). In particular they seemed to own their definitions of professionalism rather than referring to them as being externally imposed.

## Tip 9: Embrace complexity and uncertainty

Because the real world of medicine is complex and messy, facilitators may find it helpful to think of professionalism in terms of capabilities, rather than narrow competencies (Neve & Hanks 2015). Ambiguity and uncertainty are inevitable aspects of this complexity. Intolerance of these in doctors can lead to anxiety, burnout, excessive testing and treatment of patients ( Luther & Crandall 2011) and they can, similarly, cause negative reactions in medical students. We advocate dedicating time explicitly discussing uncertainty and how it can cause anxiety, frustration and risks for patients. Discussion about real life experiences in a small group can help students to ‘get’ troublesome and threshold professionalism concepts such as uncertainty, culture and holistic care ( Neve et al. 2016). It is important to recruit facilitators who are open minded and can model an ability to embrace complexity and uncertainty in their work.

## Tip 10: Be alert to, and promote learning from, diversity issues

The diversity of students within a group brings both challenges and opportunities. Students’ perceptions of professionalism will be influenced by their cultural and socio-economic backgrounds as well as their past experiences of healthcare. There may be differences, for example, in the value different students place on altruism, discipline and being accessible to patients ( Chandratilake et al. 2012). Within small groups, students with language difficulties may find active participation difficult, while a lack of cultural fit may be stressful for students and can lead to confrontation or to misunderstandings by teachers and peers who may perceive ‘different’ behaviour as unprofessional ( Jha et al. 2015). The small group learning process may also be problematic for students - for example, students who value hierarchy and acting with confidence may feel that questioning the views or behaviours of clinicians is disrespectful ( Jha et al. 2015) or be reluctant give critical feedback or admit uncertainties within their group.

Facilitators also need to be alert to gender differences. For example fewer women may volunteer to become small group leaders (Wayne et al. 2015). Facilitators need to be alert to their own unconscious biases, using approaches that do not favour a particular group and ensuring all contributions are valued equally.

Susan Cain’s book ‘Quiet’ ( 2012) challenges us to consider how the high interaction of small group discussions may disadvantage introverts, who may need time to think through their ideas before speaking. Building in pair and threesome activities can enable quieter students to better share their views. Pre or post group e-discussions boards can complement face to face discussions; it is often the written reflections of quieter students that offer the group the most powerful insights. This can help extrovert students, who may find independent reflection harder, to appreciate their quieter colleagues and see how reflection can lead to creative thought and new perspectives.

Being part of a diverse group can offer great opportunities for learning. Making time within sessions to explore and understand the complexity of others’ cultural and social attitudes and learning to appreciate and value difference are important elements of professionalism and will prepare students for work in increasingly diverse healthcare environments and teams.

## Tip 11: Encourage buy-in by communicating the purpose of groups to staff and students

*“Student expectations must correspond to those of faculty if the learning environment is to be successful”* ( Modell 1976 p.S71). Students’ expectations of professionalism learning groups may differ from that of the School and inconsistent messages from facilitators in different groups will undermine the programme. Faculty may not understand the purpose of groups and programme leads may find it hard to explain their complex nature and benefits in a few easily digestible nuggets. Both students and staff may struggle to see the relevance of the groups to the ‘biomedical’ world of medicine. If students are to value their groups as highly as their pathology lectures it is important that professionalism learning is assessed.

Publicity needs to be proactively managed. Be clear and specific about the aims of the programme and how each year differs and builds on previous years. Use student quotes (from their verbal or written reflections) to demonstrate that important learning is taking place and its relevance to clinical practice. Demonstrate the links to patient safety, clinical reasoning and communication skills. Present at grand rounds and faculty events, involve student ‘champions’ and recruit clinical facilitators from local trusts as ambassadors of the cause. Encourage the resistant to sit in and observe a session. This, in our experience, is often a perspective changing experience.

## Tip 12: Support and train your facilitators:

The role of the facilitator is not always easy. Planning sessions is difficult when you cannot predict what experiences students will share and what professionalism issues these will raise. Discussions may highlight unexpected issues or trigger strong emotions. Induction and ongoing training are vital elements of any programme and the tips above can offer a useful structure for this. Facilitators will need an understanding of basic educational theory, evidence and group dynamic issues and the opportunity to rehearse skills in a safe environment. They also need to understand there is no single right answer to any group problem. Ideally programmes will offer regular informal peer support and de-briefing with space and time to meet informally to share approaches, successes, and to develop creative ways of addressing problems. Giving facilitators session outlines which include learning goals and suggestions for challenging questions and activities is helpful, particularly for new facilitators. Observation and feedback by a senior member of faculty can be hugely beneficial for facilitators, particularly when new to their role. This can lead to useful learning for both parties.

Processes need to be in place to share student feedback with facilitators as well as the findings of, and responses to, wider programme evaluations. Facilitators are well placed to notice students who are struggling and should know who to contact if they have concerns about the group or a student or if, during group discussion, concerns are raised about a member of staff or patient safety. They need to understand raising concern and fitness to practise policies.

## Conclusion

Facilitated, small reflective groups can support professionalism learning in multiple ways, enabling students to learn from experience, explore assumptions, emotions and alternative perspectives, develop a range of vital skills and question the hidden curriculum. They offer a unique chance for faculty to role model and influence the development of students’ professional identity in a constructive and meaningful way. An important benefit is that facilitators often find themselves reflecting on their own practice in greater depth and noticing and challenging the behaviour of colleagues more readily. These changes can start to filter through an organisation, shifting attitudes to professionalism and leading to a change in culture.

## Notes On Contributors

HILARY NEVE is a General Practitioner, National Teaching Fellow and Director of the Small Group Learning, Professionalism and Social Engagement programmes at Plymouth University Peninsula Medical School, UK.

RACHEL MORRIS is a General Practitioner, Executive Coach and Trainer and Professionalism Lead at the School of Clinical Medicine, University of Cambridge, UK.

## References

[ref1] Baernstein A. & Fryer-Edwards K. (2003). Promoting reflection on professionalism: a comparison trial of educational interventions for medical students. Academic Medicine. 78(7),742–747. 10.1097/00001888-200307000-00018 12857697

[ref2] Birden H. Glass N. Wilson I. Harrison M. Usherwood T. & Nass D. (2013). Teaching professionalism in medical education: a Best Evidence Medical Education (BEME) systematic review. BEME Guide No. 25. Medical teacher. 35(7),e1252–e1266. 10.3109/0142159X.2013.789132 23829342

[ref3] Birden H. Usherwood T. (2013). They liked it if you said you cried: how medical students perceive the teaching of professionalism. Med J Aust. 199(6),40–9. 10.5694/mja12.11827 24033214

[ref4] Cain S . 2012. Cain, S.(2012). Quiet: The Power of Introverts in a World That Can’t Stop Talking. New York: Crown Publishers.

[ref5] Chandratilake M. McAleer S. & Gibson J. (2012). Cultural similarities and differences in medical professionalism: a multi‐region study. Medical education. 46(3),257–266. 10.1111/j.1365-2923.2011.04153.x 22324525

[ref6] Cohen J. J. (2006). Professionalism in medical education, an American perspective: from evidence to accountability. Medical education. 40(7),607–617. 10.1111/j.1365-2929.2006.02512.x 16836532

[ref7] Cruess R. L. Cruess S. R. Boudreau J. D. Snell L. & Steinert Y. (2014). Reframing medical education to support professional identity formation. Academic Medicine. 89(11),1446–1451. 10.1097/ACM.0000000000000427 25054423

[ref8] Daloz L.A. (1986). Effective teaching and mentorship: realizing the transformational power of adult learning experiences. San Francisco: Jossey-Bass.

[ref9] Dyche L. Epstein R. M. (2011). Curiosity and medical education. Medical education. 45(7),663–668. 10.1111/j.1365-2923.2011.03944.x 21649698

[ref10] Epstein R. M. & Hundert E. M. (2002). Defining and assessing professional competence. Jama. 287(2),226–235. 10.1001/jama.287.2.226 11779266

[ref11] Feudtner C. Christakis D. A. & Christakis N. A. (1994). Do clinical clerks suffer ethical erosion? Students’ perceptions of their ethical environment and personal development. Academic medicine. 69(8),670–9. 10.1097/00001888-199408000-00017 8054117

[ref12] Fraser S. W. & Greenhalgh T. (2001). Coping with complexity: educating for capability. BMJ: British Medical Journal. 323(7316),799–803. 10.1136/bmj.323.7316.799 11588088 PMC1121342

[ref13] Greenhalgh T. & Hurwitz B. (1999). Why study narrative? BMJ: British Medical Journal. 318(7175),48–50. 10.1136/bmj.318.7175.48 9872892 PMC1114541

[ref14] Guillemin M. & Gillam L. (2015). Emotions, narratives, and ethical mindfulness. Academic Medicine. 90(6),726–731. 10.1097/ACM.0000000000000709 25853684

[ref15] Hafferty F. W. (1998). Beyond curriculum reform: confronting medicine’s hidden curriculum. Academic Medicine. 73(4),403–7. 10.1097/00001888-199804000-00013 9580717

[ref16] Hodges B.D. Ginsburg S. Cruess R. Cruess S. Delport R. Hafferty F. Ho M. Holmboe E. Hotman M. Ohbu S. Rees C. Cate O.T. Tsuawa Y. Van Mook W. Wilkinson T. Wade W. (2011). Assessment of professionalism: Recommendations from the Ottawa 2010 Conference. Medical Teacher. 33(5):354–363. 10.3109/0142159X.2011.577300 21517683

[ref17] Ho C. H. (2015). Slowing Medical Education Through Schwartz Center Rounds. Academic Medicine. 90(10),1290. 10.1097/ACM.0000000000000865 27002875

[ref18] Howe A. (2002). Professional development in undergraduate medical curricula-the key to the door of a new culture? Medical education. 36(4),353–359. 10.1046/j.1365-2923.2002.01168.x 11940176

[ref19] Jha V. Mclean M. Gibbs T. J. & Sandars J. (2015). Medical professionalism across cultures: A challenge for medicine and medical education. Medical teacher. 37(1),74–80. 10.3109/0142159X.2014.920492 25073712

[ref20] Kellett J. Papageorgiou A. Cavenagh P. Salter C. Miles S. & Leinster S. J. (2015). The preparedness of newly qualified doctors-Views of Foundation doctors and supervisors. Medical teacher. 37(10),949–954. 10.3109/0142159X.2014.970619 25308805

[ref21] Luther V. P. & Crandall S. J. (2011). Commentary: ambiguity and uncertainty: neglected elements of medical education curricula? Academic Medicine. 86(7),799–800. 10.1097/ACM.0b013e31821da915 21715991

[ref22] Mann K. Gordon J. & MacLeod A. (2009). Reflection and reflective practice in health professions education: a systematic review. Advances in health sciences education. 14(4),595. 10.1007/s10459-007-9090-2 18034364

[ref23] Midmer D. (2002). The processing cycle. BMJ. 325(7371):S140–S140. 12411385 10.1136/bmj.325.7371.s140

[ref24] Modell H. (1996). Preparing students to participate in an active learning environment. Advan Physiol Educ. 270,69–77. 10.1152/advances.1996.270.6.S69 8712256

[ref25] Monrouxe L. V. Rees C. E. & Hu W. (2011). Differences in medical students’ explicit discourses of professionalism: acting, representing, becoming. Medical education. 45(6),585–602. 10.1111/j.1365-2923.2010.03878.x 21564198

[ref26] Monrouxe L. Bullock A. D. Rees C. Mattick K. Webb K. L. Lall K. & Lundin R. (2015). Foundation doctors, transitions and emotions: final report to the GMC: July 2015. www.gmc-uk.org

[ref27] Neve H Collett T. (2014). Revealing the hidden curriculum to medical students: insights from threshold concept theory. Fifth International Biennial Threshold Concepts Conference, Threshold Concepts in Practice, Durham: UK. Available from http://www.ee.ucl.ac.uk/~mflanaga/abstracts/TC14Abstract32.pdf

[ref28] Neve H. Lloyd H. & Collett T. (2017). Understanding students’ experiences of professionalism learning: a ‘threshold’ approach. Teaching in Higher Education. 22(1),92–108. 10.1080/13562517.2016.1221810

[ref29] Neve H. Hanks S. (2016). When I say.. capability. Medical education. 50(6),610–611. 10.1111/medu.12956 27170079

[ref30] Norman G. (2009). Teaching basic science to optimize transfer. Medical Teacher. 31(9),807–811. 10.1080/01421590903049814 19811185

[ref31] Passi V. Doug M. Peile J. T. & Johnson N. (2010). Developing medical professionalism in future doctors: a systematic review. International journal of medical education. 1,19. 10.5116/ijme.4bda.ca2a

[ref32] Pinsky L. Robins L. & Benlzzy J. (2004). The story in medicine: skills for MDs. Medical education. 38(11),1200–1201. 10.1111/j.1365-2929.2004.02004.x 15507037

[ref33] Platzer H. Blake D. & Ashford D. (2000). Barriers to learning from reflection: a study of the use of groupwork with post‐registration nurses. Journal of advanced nursing. 31(5),1001–1008. 10.1046/j.1365-2648.2000.01396.x 10840232

[ref34] Prince M. (2004). Does active learning work? A review of the research. Journal of engineering education. 93(3),223–231. 10.1002/j.2168-9830.2004.tb00809.x

[ref35] Rabow M. W. Remen R. N. Parmelee D. X. & Inui T. S. (2010). Professional formation: extending medicine’s lineage of service into the next century. Academic Medicine. 85(2),310–317. 10.1097/ACM.0b013e3181c887f7 20107361

[ref36] Rosenbaum M. E. Ferguson K. J. & Herwaldt L. A. (2005). In their own words: presenting the patient’s perspective using research‐based theatre. Medical education. 39(6),622–631 10.1111/j.1365-2929.2005.02181.x 15910439

[ref37] Salmon P. & Young B. (2011). Creativity in clinical communication: from communication skills to skilled communication. Medical education. 45(3),217–226. 10.1111/j.1365-2923.2010.03801.x 21299597

[ref38] van Mook W. N. de Grave W. S. Wass V. O’Sullivan H. Zwaveling J. H. Schuwirth L. W. & van der Vleuten C. P. (2009). Professionalism: Evolution of the concept. European Journal of Internal Medicine. 20(4),e81–e84. 10.1016/j.ejim.2008.10.005 19524164

[ref39] Wald H. S. (2015). Professional identity (trans) formation in medical education: reflection, relationship, resilience. Academic Medicine. 90(6),701–706. 10.1097/ACM.0000000000000731 25881651

[ref40] Wayne N. L. Vermillion M. & Uijtdehaage S. (2010). Gender differences in leadership amongst first-year medical students in the small-group setting. Academic Medicine. 85(8),1276–1281. 10.1097/ACM.0b013e3181e5f2ce 20671452

[ref41] Wood D. F. (2016). Mens sana in corpore sano: student well‐being and the development of resilience. Medical education. 50(1),20–23 10.1111/medu.12934 26695463 PMC5064634

