# Supplementary material for: Twelve tips for promoting professionalism through reflective small group learning
Source: MedEdPublish (2016). 2017 Mar 6;6:43. [Version 1] doi: 10.15694/mep.2017.000043 (PMC10885239; doi:10.15694/mep.2017.000043)
Supplement: Peer review history

# Reviewer report: Reviewer response for version 1

**DOI:** 10.21956/mep.19451.r28648

**Published:** 2021-02-02

**Authors:** Annwyne Houldsworth

**Affiliations:** 1 HECL

This review has been migrated. The reviewer awarded 5 stars out of 5 Thank you for compiling this extremely helpful and insightful paper, detailing some very poignant issues surrounding professionalism curricular in medical education. I recommend this publication as a treasure trove of advice for acquiring the required skills for small group practice (eg. PBL) in general and professionalism groups in particular.There is a great deal of perceptive advice offered by the authors, describing the importance of environment, collaboration, narratives, reflection and moral concerns.The instigation of ‘dedicated pauses’ is such a valuable tool to encourage participation and engagement; however, I would like to add that some students take longer to reflect on an issue so a further suggestion is to include a short period of time at the beginning of the next group session for those who have further thoughts to share.The discussion about issues of diversity was particularly relevant in an integrated group of students, on both a cultural and interprofessional level, where the use of smaller ‘breakout groups’ is such a useful tool to encourage engagement from the otherwise ‘quiet students’. This is also possible during online digital conference type sessions allowing students to break into smaller groups and report back to the main group, highly relevant in the current global pandemic.Exploring individual culture and social attitudes as personal narratives can be highly informative, developing empathy with new perspectives and attitudes as well as developing their storytelling skills. The paper also suggested the idea of ‘learning walks’ to promote the recruitment of facilitators by sitting in and observing groups in action. I appreciate the optimism of the authors in their hope for changes in professionalism where best practice can filter through the organisation, leading to a change in its culture.The authors’ comments on ‘medical culture’ and unprofessional behaviour are both valid and important contributions and are often perceived as ‘the elephant in the room’. In addition to clinical professionalism, the professional behaviours of the facilitator, modelling the attributes to be demonstrated is a key factor where training to support this may be required. The modelling of professionalism within our own workplace, clinical or academic, including the relationships and treatment of other staff members should be considered, promoting values of fair play and mutual support must be more than a façade or theoretical rhetoric but integral in the workplace, to be observed by the students. Students can be very astute about their learning environment and may respond negatively to hypocrisy from their mentors. Indeed, the academic environment is not the best example to demonstrate many of the professionalism principles and may be unconsciously incompetent to the values detailed in this excellent paper.As a final point, could there some dissonance between the confidentiality of sharing a student’s personal related experiences in the group and reporting concerns about a student to another colleague who is struggling as, clearly, discernment and wisdom must be applied? Thank you so much for highlighting these 12 enlightening tips in medical education.

Reviewer Expertise:

NA

No decision status is available

# Reviewer report: Reviewer response for version 1

**DOI:** 10.21956/mep.19451.r28646

**Published:** 2019-01-30

**Authors:** Yukihiro Ikeda

**Affiliations:** 1 yuyu@med.kindai.ac.jp

This review has been migrated. The reviewer awarded 4 stars out of 5 Education of professionalism is also becoming important in the field of medical education in Japan. This paper was written in practice, how to do it to make education of professionalism fulfilling, it was very helpful. Especially Tip 12: Support and train your facilitators: was able to sympathize.

Reviewer Expertise:

NA

No decision status is available

# Reviewer report: Reviewer response for version 1

**DOI:** 10.21956/mep.19451.r28651

**Published:** 2018-06-06

**Authors:** Anthony David.M

**Affiliations:** 1 Soho

This review has been migrated. The reviewer awarded 3 stars out of 5 A very useful article contributing to medical education indeed. The use of small group teaching and learning methods is active, participatory and useful. Reflection on the part of medical students helps them mature in their thinking. The tips given, all twelve of them are practical and useful to faculty in medical education. Thank you authors.

Reviewer Expertise:

NA

No decision status is available

# Reviewer report: Reviewer response for version 1

**DOI:** 10.21956/mep.19451.r28644

**Published:** 2017-03-11

**Authors:** Lakshmi Nagappa Naik

**Affiliations:** 1 Melaka Manipal Medical College

This review has been migrated. The reviewer awarded 5 stars out of 5 Very well thought of tips which enable users to implement the teaching methodologies with ease . reflective practice should be more reiterated as a basis through all clinical education and embedding humanity and art along with narrative medicine ,for which there should be a recognised mandatory activity . Portfolio based learning too needs to be emphasised along with mentorship , which ensue positive role modelling .

Reviewer Expertise:

NA

No decision status is available

# Reviewer report: Reviewer response for version 1

**DOI:** 10.21956/mep.19451.r28650

**Published:** 2017-03-08

**Authors:** sathyanarayanan varadarajan

**Affiliations:** 1 SRM Medical College, SRM University

This review has been migrated. The reviewer awarded 4 stars out of 5 In this interesting article, the authors share lessons learnt from a total of 18 years’ experience of leading professionalism small group learning programmes in two very different UK medical schools. They discuss twelve tips for promoting professionalism through reflective small group learning. The tips are designed to be useful for programme leads and small group facilitators involved in medical education. The authors feel that Learning effectively from one’s experience is critical in developing and maintaining lifelong competence, particularly learning professionalism in medicine, which is considered as “an extraordinarily complex phenomenon”. They observed that Students value sharing their experiences in groups, recognizing that exploring complex real life situations rather than the “superficial or trivial’ scenarios used in didactic teaching sessions, can be the best way to learn professionalism.They suggest Reflecting within facilitated small groups, rather than alone, can be particularly effective in learning professionalism. Small groups also can help students develop vital interpersonal and workplace skills. The other advantages are, Small groups can provide mutual support, offer information from multiple perspectives, and challenge students to consider things more deeply. Another important benefit is that facilitators often find themselves reflecting on their own practice in greater depth and noticing and challenging the behavior of colleagues more readily. These changes can start to filter through an organization, shifting attitudes to professionalism and leading to a change in culture.After discussing each tips, the authors conclude that Facilitated, small reflective groups can support professionalism learning in multiple ways, enabling students to learn from experience, explore assumptions, emotions and alternative perspectives, develop a range of vital skills and question the hidden curriculum. They offer a unique chance for faculty to role model and influence the development of students’ professional identity in a constructive and meaningful way.

Reviewer Expertise:

NA

No decision status is available

# Reviewer report: Reviewer response for version 1

**DOI:** 10.21956/mep.19451.r28645

**Published:** 2017-03-07

**Authors:** Jill Thistlethwaite

**Affiliations:** 1 UTS

This review has been migrated. The reviewer awarded 4 stars out of 5 This is a very interesting paper on a complex issue. A further tip would be to ensure that students are well prepared for this type of small group work with an orientation to its purpose and importance. Students should be encouraged to set their ground rules for the group (which they may have done for previous group work but important to revisit). Students need to feel safe when discussing potentially difficult areas; they need to trust their group members in terms of confidentiality; they need to know what a facilitator may do if examples are brought to the group that need further action outside the group. There should also be ground rules for e-discussion and what may be uploaded - confidentiality here is important and who moderates the discussion. In terms of role modelling, students may wish to know if similar groups are run for faculty/clinical teachers to reflect on professionalism as part of CPD.

Reviewer Expertise:

NA

No decision status is available

# Reviewer report: Reviewer response for version 1

**DOI:** 10.21956/mep.19451.r28649

**Published:** 2017-03-07

**Authors:** Gert Van Zyl

**Affiliations:** 1 University of the Free State

This review has been migrated. The reviewer awarded 4 stars out of 5 What an enjoyable paper. To the point, with a focus and practical approach. Professionalism and the student was never lost in the paper. To present the research in a format of “tips” was a clever strategy and very useful. The approach to teaching professionalism was clear and to the point and made a very valuable contribution to the topic. The vision to infuse professionalism by allowing students to share and reflect from own experience is not only creative, but provides opportunities to understand and instill ownership for maintaining professionalism as part of a change in culture.

Reviewer Expertise:

NA

No decision status is available

# Reviewer report: Reviewer response for version 1

**DOI:** 10.21956/mep.19451.r28643

**Published:** 2017-03-06

**Authors:** Richard Hays

**Affiliations:** 1 James Cook University

This review has been migrated. The reviewer awarded 4 stars out of 5 This paper presents a useful summary of the key issues in teaching professionalism in a more interactive and meaningful way. I would have liked to see role modelling in Tip 12, as tutors may have important roles in ensuring that differences are respected and professional behaviours encouraged within group discussions.training to ensure that challenging issues are discussed (Tip 10) in a way that acknowledges complexity and uncertainty (Tip 9). The process may be just as important as the content - I think this is the point the authors make in Tip 8 - as learners must come to accept and internalise constructs of prrofessionalism.

Reviewer Expertise:

NA

No decision status is available

# Reviewer report: Reviewer response for version 1

**DOI:** 10.21956/mep.19451.r28647

**Published:** 2017-03-06

**Authors:** Trevor Gibbs

**Affiliations:** 1 AMEE

This review has been migrated. The reviewer awarded 4 stars out of 5 I very much enjoyed reading this paper, it was clear, concise and provide twelve very appropriate tips to faculty concerned with such a difficult subject as professionalism.Although I think that the authors partly explored it in Tips 9 and 12, I think the issue of non-professionalism needs to be explored. We have seen some of the bad effects from "whistle-blowing" in the NHS, of students observing non-professional behaviour, of poor role-models and to observing medical mistakes. Whilst these are thankfully rather rare, perhaps it is time we explored with students how to deal with these issues

Reviewer Expertise:

NA

No decision status is available
